# Dome-shaped macula with protrusion of inner part of sclera

**DOI:** 10.1016/j.ajoc.2023.101926

**Published:** 2023-09-04

**Authors:** Tomonari Takahashi, Tae Igarashi-Yokoi, Noriko Nakao, Kengo Uramoto, Takeshi Yoshida, Kyoko Ohno-Matsui

**Affiliations:** Department of Ophthalmology and Visual Science, Tokyo Medical and Dental University, 1-5-45 Yushima, Bunkyo-ku, Tokyo, 113-8510, Japan

**Keywords:** DSM, High myopia, Intrascleral blood vessel, MNV, Retina, Optical coherence tomography

## Abstract

**Purpose:**

To report our findings in a patient with a two layered dome shaped macula (DSM) in which only the inner layer of the sclera protruded anteriorly.

**Observations:**

An 84-year-old woman with high myopia had a DSM in both eyes. The optical coherence tomographic (OCT) image of the left eye showed a uniform thickening of the foveal sclera, but the DSM of the right eye was split into an inner and outer layer by intrascleral blood vessels running between the two layers. OCT showed that only the inner layer of the sclera protruded anteriorly while the outer layer remained in its normal position.

**Conclusions and importance:**

The two layered DSM suggests that the etiology of DSMs may be more complex.

## 1 Introduction

A dome-shaped macula (DSM) is a morphological shape of the posterior fundus of the eye, and it was first reported by Gaucher and associates as a finding in highly myopic eyes.^1^ It was characterized by a convex elevation of the macula seen in optical coherence tomographic (OCT) images.^1^ A DSM has been reported to occur in 10.7–12% of highly myopic eyes in hospital-based patients,^1^, ^2^, ^3^ and it was identified by the presence of an inward bulge of the retinal pigment epithelium (RPE) of more than 50 μm above a simulated line tangent to the outer surface of RPE at the base of a posterior staphyloma.^4^^,^^5^

Imamura and associates^6^ reported that a DSM developed from a sub-foveal localized thickening of the sclera although the mechanism causing the thickening was not reported. We report our findings in a highly myopic eye with a DSM in which the DSM was split into an inner and outer layer by intrascleral blood vessels between them. Only the inner part of sclera protruded anteriorly.

### 1.1 Case report

An 84-year-old woman did not have any family history of ocular or systemic diseases. She also did not have a history of ocular trauma, ocular surgical procedures, uveitis, and connective tissue disorders.

She received an intravitreal ranibizumab (Lucentis, Novartis Farmaceutica, Inc) injection three times for a myopic macular neovascularization (MNV) in her right eye at the age of 74 years. She also received an intravitreal injection of ranibizumab once for the MNV in her left eye at the age of 79 years. The MNVs were resolved in both eyes although the MNV eventually progressed to macular atrophy. The vision of her right eye before beginning the CNVM treatment was 20/40, and it improved to 20/20 after the treatment. The MNV eventually progressed to macular atrophy, and the final vision was 20/200.

At our initial examination, her best-corrected visual acuity (BCVA) was 20/100 in the right eye and 20/25 in the left eye. The axial length was 27.41 mm in the right eye and 28.17 mm in the left eye. Slit-lamp examinations showed that the anterior segment was within normal limits in both eyes with implanted intraocular lenses. Fundus examinations showed a myopic conus in both eyes, macular atrophy in the right eye and patchy atrophy in the left eye (Fig. 1A). The OCT images showed ridge-shaped DSMs in both eyes which were more obvious in the vertical OCT sections. The right eye had a scarred MNV and MNV-related macular atrophy, and the left eye had a small patchy atrophy superior to the macula.

**Fig. 1:**
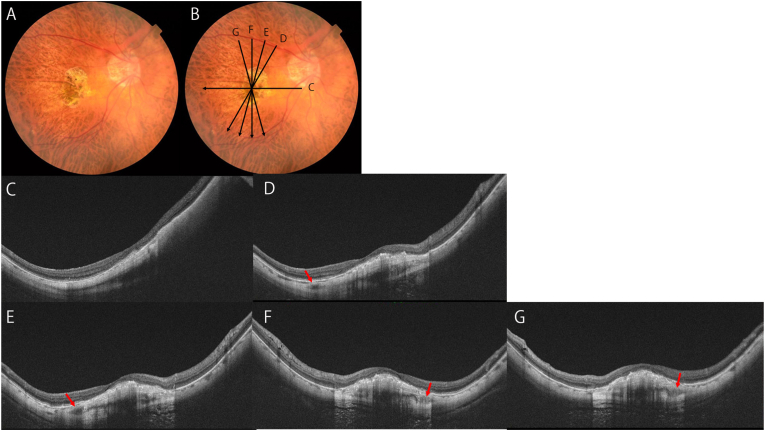
Dome-shaped macula accompanying with splitting of foveal scleral tissues due to intrascleral blood vessels. Observations of the DSM in a highly myopic eye in radial scan images obtained by swept-source OCT. A. Right fundus of an 84 year-old woman with axial length of 27.41mm shows macular atrophy due to myopic macular neovascularization (MNV). She had anti-VEGF therapies three times. Scan lines are OCT sections in the images C to G. B. OCT images show dome-shaped macula (DSM) as well as scarred MNV are observed. Cross sections of intrascleral vessels are observed (arrows). Careful observation shows that sclera is split into two layers at the depth of intrascleral blood vessels. The inner part of sclera alone seems to be protruded anteriorly while the outer part is not thickened or protruded.

The DSM of the right eye was split into an inner and an outer layer by large intrascleral vessels running between them. In this split sclera case with DSM, we were able to find intrascleral vessels in the superior (Fig. 1D and E) and inferior (Fig. 1F and G) areas of the macula in the OCT B scan serial images. Between the two intrascleral blood vessels, there was a spindle and planate shaped low-density area which split the sclera into the inner and outer layers. In contrast, the splitting of the sclera was not obvious in the areas outside the macula.

A careful examination of the OCT images showed that only the inner layer of the sclera protruded anteriorly while the outer layer remained in its normal position (Fig. 1C–G). In the left eye, analysis of the OCT volume scan reveals that the intrascleral hyporeflective spaces were not due to scleral splitting, but rather mimicry due to intrascleral vasculature that were incidentally imaged parallel to the vessels. (Supplemental Fig. 1).

## 2 Discussion

Imamura et al.^6^ used EDI-OCT images to show that a DSM was a focal thickening of the sclera in the macular area. However, the pathogenesis of the thickening of the foveal sclera was not definitively determined. In our patient, the left eye had a uniform thickening of the foveal sclera (Supplemental Fig. 1). However, the sclera in the right eye was split into an inner layer and an outer layer by large intrascleral vessels. Then, only the inner layer protruded anteriorly while the outer layer remained in its normal position.

Ohno-Matsui et al.^7^ analyzed the intrascleral blood vessels in highly myopic eyes using swept-source OCT images. They found that these intrascleral vessels were branches of the short posterior ciliary arteries (SPCAs), long posterior ciliary arteries (LPCAs), and macular vortex veins. Cross sections of the intrascleral blood vessels that were seen in our patient were also present in their study. In the areas where the cross sections were seen, the surrounding scleral fibrotic tissues were pushed outward by the blood vessels and the sclera was stretched into a rhomboid shape. Based on their findings, we suggest that the splitting of the scleral tissue around the intrascleral blood vessels may progress to a large dehiscence between the inner and outer layers of the sclera as was seen in our patient.

Ohno-Matsui et al.^7^ also reported that the sclera could be split into an inner and outer layer in a large area of the posterior fundus. Our findings showed that the scleral split was detected only around the area where the intrascleral vessels could be observed. However, we suggest that the sclera can be split into two layers in areas without intrascleral vessels based on the results of the left eye and the study by Ohno-Matsui et al.^7^ Fang et al.^8^ reported that Bruch's membrane (BM) defects around the macula reduced the tension on the sclera and may have contributed to the development of the DSMs. In our patient, BM defects in the atrophic area may have contributed to a more prominent DSM.

Ishida et al.^9^ reported that MNVs had blood flow in the active, scarred, and atrophic phases. The scleral perforating vessels originated from the short posterior ciliary arteries ran through the defects of Bruch's membrane. Giuffre et al.^10^ reported that the perforating scleral vessels were often in the areas of MNVs and may play a role in the development of the MNVs. The MNVs could get larger from the perforating vessels and this could affect the changes in the scleral structure.

Our patient had a history of MNVs in both eyes, and there were also BM defects in both eyes. However, only the right eye had a split sclera. The difference between the two eyes was that the intrascleral vessels at the edges of the DSM was present only in the right eye. A comparison of the two eyes suggested that the split sclera was caused by the intrascleral vessels.

The existence and the location of the intrascleral blood vessels and scleral tissues splitting around intrascleral vessels may have affected the formation and shape of the DSM. These findings suggest that the pathogenesis and the progression of DSMs may be diverse and complex.

## 3 Conclusions

A DSM is characterized by a convex elevation of the macula. The pathogenesis of a DSM is still not definitively determined but some studies have reported that a DSM was accompanied by a focal thickening of the sclera. We report a unique case of DSM in which the sclera was split into an inner and outer layer by intrascleral blood vessels.

## Funding

No funding was provided to do this project.

## Authorship

All authors attest that they meet the current ICME criteria for Authorship.

## Patient consent

Written consent to publish this case has not been obtained. This report does not contain any personal identifying information.

## Declaration of competing interest

The authors declare that they have no known competing financial interests or personal relationships that could have appeared to influence the work reported in this paper.The authors have no conflict of interest.

## Figures and Tables

**figs1:**
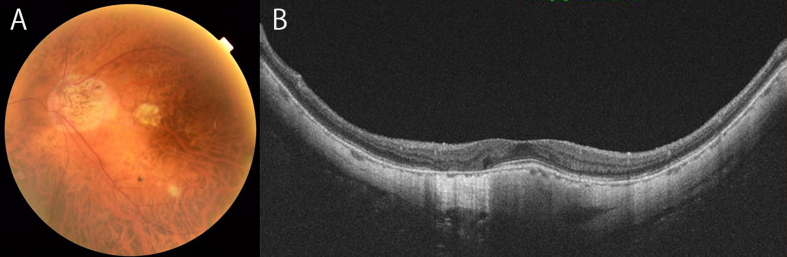
Dome-shaped macula with uniform thickening of foveal sclera in the other eye of Fig. 1. A. Left fundus of an 84 year-old woman with axial length of 28.17mm shows patchy atrophy. She had anti-VEGF therapies one time. B. Vertical scan of OCT image shows uniform thickening of foveal sclera. There is an intrascleral blood vessel but scleral split does not exist.
